# Malignant currents: sodium leak channel NALCN propels prostate cancer aggressiveness

**DOI:** 10.15252/embj.2023114986

**Published:** 2023-08-28

**Authors:** Ioana Stejerean‐Todoran, Ivan Bogeski

**Affiliations:** ^1^ Molecular Physiology, Institute of Cardiovascular Physiology, University Medical Center Georg‐August‐University Göttingen Germany

**Keywords:** Cancer, Cell Adhesion, Polarity & Cytoskeleton, Signal Transduction

## Abstract

Although ion transporters and channels have been extensively studied over the last couple of decades, there are still unresolved aspects with regards to their contribution to cancer cell biology. Recent work by Folcher *et al* (2023) reports a critical role for Na^+^ leak channel NALCN in metastatic prostate cancer. The study demonstrates that NALCN promotes metastatic spread to distant organs by controlling Ca^2+^ signaling.

## Graphical abstract

Recent work uncovers a functional role for Na^+^ leak channel NALCN in prostate cancer, driving Ca^2+^‐dependent invasive cell remodeling and metastasis.

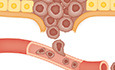

Albeit underinvestigated for a long time, the role of ion channels and transporters in cancer cell pathobiology is becoming increasingly apparent. In this context, Na^+^, K^+^, and Ca^2+^ signals have been implicated in controlling various cancer cell functions (Leanza *et al*, 2016; Prevarskaya *et al*, 2018; Vultur *et al*, 2018).

Prostate cancer is among the most prevalent malignancies in men and has a high fatality rate due to its tendency to metastasize to distant organs, commonly to the bones. As in many other cancer entities, prostate cancer cell biology is controlled by various ion channels. In fact, the importance of Ca^2+^ signaling has been reported over 40 years ago. It was initially believed that Ca^2+^ diminishes tumor aggressive phenotype; however, following studies showed that Ca^2+^ may not only be a *foe* but also a *friend* of cancer progression. These initial studies illustrate that further knowledge is needed in order to understand the importance of Ca^2+^ in prostate cancer. Store‐operated Ca^2+^ entry (SOCE), for example, is one of the major Ca^2+^ access routes into cells and is thus involved in cellular functions associated with cancer aggressive behavior, including invasion. Store‐operated Ca^2+^ entry is regulated by members of the ORAI and STIM families. ORAI channels are plasma membrane‐bound Ca^2+^‐selective ion channels, while STIM proteins reside in the ER membrane (Prakriya & Lewis, 2015). Indeed, previous studies have demonstrated that SOCE is altered in prostate cancer cells (Holzmann *et al*, 2013, 2015; Dubois *et al*, 2014; Perrouin‐Verbe *et al*, 2019). The mitochondrial Ca^2+^ uniporter (MCU) complex is tightly interconnected with SOCE by taking up Ca^2+^ ions from the IP_3_ receptors as well as by modulating SOCE through mitochondrial metabolic alterations. The importance of the SOCE–MCU axis has been recently demonstrated in melanoma (Gross *et al*, 2022; Stejerean‐Todoran *et al*, 2022).

In addition to Ca^2+^ signals, alterations in Na^+^ hemostasis are also indicative of aggressive disease (reviewed in Horne *et al*, 2021). The Na^+^ leak channel NALCN, first reported in hippocampal neurons (Lu *et al*, 2007), is a membrane multiprotein complex involved in cell excitability and trafficking whose genetic modifications are associated with neurological disorders (Rahrmann *et al*, 2022). Additionally, it has been shown that NALCN expression is altered in several cancer types (reviewed in Cochet‐Bissuel *et al*, 2014). In this study, Folcher *et al* (2023) observed an upregulation of NALCN in prostate cancer and associated metastatic tumor tissues compared with normal prostate tissues, and this correlated with the expression of Src, a tyrosine kinase that stimulates cancer invasion by interacting with matrix metalloproteinases and several proteins associated with cancer cell aggressive behavior. NALCN downregulation further suppressed invadopodia formation and abolished prostate cancer cell invasion but not proliferation. Furthermore, the authors showed that the formation of invadopodial protein puncta occurs at sites of cytosolic Ca^2+^ wave initiation, which also coincide with high NALCN expression patterns in metastatic prostate cancer cells. The authors further demonstrated that cytosolic Ca^2+^ oscillations were decreased upon NALCN downregulation as well as upon the reduction of extracellular Na^+^ concentration, proving that Ca^2+^ fluctuations are dependent on continuous Na^+^ flow. Moreover, Folcher *et al* (2023) showed that NALCN‐silenced prostate cancer cells display reduced SOCE and, naturally, diminished Na^+^ flow, making the connection between NALCN‐mediated Na^+^ influx and cytosolic Ca^2+^ levels. They propose a mechanism for the maintenance of cytosolic Ca^2+^ oscillations based on the positive feedback between NALCN and the reverse Na^+^/Ca^2+^ exchanger. While in gastrointestinal cancer, loss of NALCN enhances cancer metastasis (Rahrmann *et al*, 2022). Folcher *et al* (2023) show that in prostate cancer, the high NALCN levels lead to a more aggressive phenotype *in vivo*. Namely, they prove that NALCN is upregulated in PTEN/p53 knockout mouse tumors. Furthermore, they demonstrate that the bone tissue destruction following the inoculation of low NALCN‐expressing metastatic prostate cancer cells into the tibia of immunodeficient mice is reduced compared to the control high NALCN‐expressing group and that intracardiac injection of NALCN‐overexpressing metastatic prostate cancer cells leads to the development of osteolytic lesions (Fig 1).

**Figure 1:**
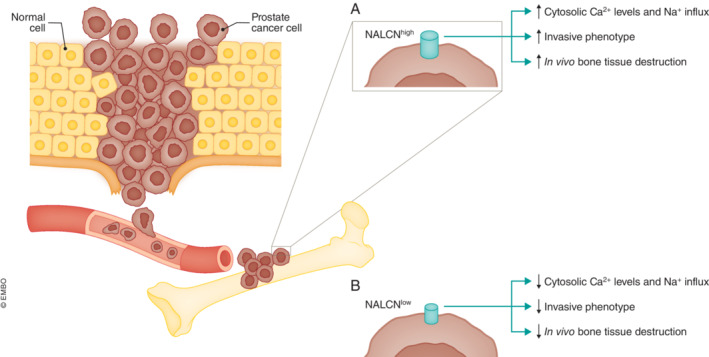
Effects of NALCN bioavailability in prostate cancer cells Prostate cancer cells display high levels of NALCN and commonly invade the bone tissue. (A) When NALCN levels are upregulated (NALCN^high^), cytosolic Ca^2+^ levels and Na^+^ influx are enhanced, resulting in a highly invasive phenotype followed by bone tissue destruction. (B) Conversely, when NALCN levels are diminished (NALCN^low^), so are cytosolic Ca^2+^ levels and Na^+^ influx, leading to a less aggressive phenotype.

The data presented in this study suggest that NALCN should receive more clinical focus in the early detection of metastatic prostate cancer. However, these findings are of broad importance and should be confirmed in a larger panel of metastatic prostate cancer cell lines. Forthcoming research exploring the functional coupling between NALCN‐mediated Na^+^ flow and Ca^2+^ fluctuations in other deadly human cancer entities will provide a better understanding of their therapeutic potential.

## Disclosure and competing interests statement

The authors declare that they have no conflict of interest.
